# Socioeconomic determinants are associated with the utilization and outcomes of active surveillance or watchful waiting in favorable‐risk prostate cancer

**DOI:** 10.1002/cam4.5650

**Published:** 2023-02-02

**Authors:** Da Huang, Xiaohao Ruan, Jingyi Huang, Ning Zhang, Guangliang Jiang, Yi Gao, Danfeng Xu, Rong Na

**Affiliations:** ^1^ Department of Urology, Ruijin Hospital Shanghai Jiao Tong University School of Medicine Shanghai China; ^2^ Department of Surgery, LKS Faculty of Medicine The University of Hong Kong Hong Kong SAR China

**Keywords:** marital status, prostatic neoplasms, rural population, watchful waiting

## Abstract

**Background:**

Active surveillance/watchful waiting (AS/WW) is feasible and effective for favorable‐risk prostate cancer (PCa). Understanding socioeconomic determinants of AS/WW may help determine the target population for social support and improve cancer‐related survival.

**Methods:**

The Surveillance, Epidemiology, and End Results Prostate with Watchful Waiting Database 18 Registries identified 229,428 adult men diagnosed with primary localized PCa (clinical T1‐T2c, N0M0) during a median follow‐up of 45 months between 2010 and 2016. Socioeconomic determinants included socioeconomic status (SES) tertiles, marital status (unmarried vs married), and residency (urban vs rural). Multivariable logistic regression and Cox models determined the adjusted odds ratios (aOR) for AS/WW utilization, and adjusted hazard ratio (aHR) for cancer‐specific survival (CSS) and overall survival (OS). The extent of missing data was evaluated by multiple imputation. Sensitivity analyses were performed in multiple imputation datasets.

**Results:**

Unmarried patients were more likely to receive AS/WW in low‐risk group (aOR, 1.20 [95%CI, 1.12–1.28]; *p* < 0.001) and favorable intermediate‐risk group (aOR, 1.41 [95%CI, 1.26–1.59]; *p* < 0.001) than married patients. Urban patients had 0.77‐fold lower likelihood of AS/WW than rural patients in low‐risk group (95% CI, 0.68–0.87; *p* < 0.001), but not in favorable intermediate‐risk groups. Among patients undertaking AS/WW, a significantly worse OS was observed among unmarried patients comparing to married group (aHR, 1.98 [95% CI, 1.50–2.60]; *p* < 0.001), and patients with high SES had better CSS than low group (aHR, 0.08 [95%CI, 0.01–0.69]; *p* = 0.02). No significant survival difference was found between urban and rural patients.

**Conclusions and Relevance:**

Unmarried or urban patients had significantly higher rates of AS/WW. The utilization and efficacy of conservative management were affected by socioeconomic factors, which might serve as a barrier of treatment decision‐making and targeted a population in need of social support.

## Graphical abstract

In this population‐based, cross‐sectional study, the utilization rates of conservative management were significantly higher in low socioeconomic status (SES), unmarried and urban patients diagnosed with low‐risk prostate cancer. Unmarried status and low SES were associated with worse overall survival among patients undertaking active surveillance/watchful waiting. The utilization of conservative management was affected by socioeconomic factors, which might serve as a barrier of treatment decision‐making and targeted a population in need of social support.

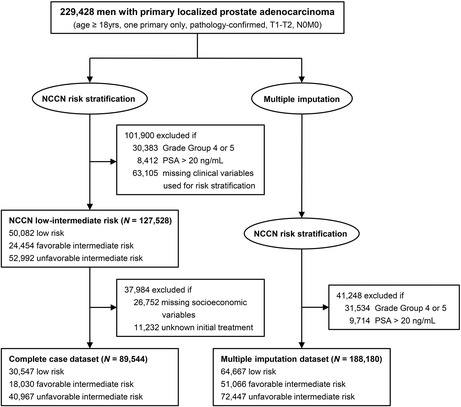

**Key points**

*Question*: Do the socioeconomic factors influence the utilization and outcomes of active surveillance or watchful waiting (AS/WW) among prostate cancer (PCa) patients?

*Findings*: In this population‐based, observational cohort study, the utilization rates of conservative management were significantly higher in low socioeconomic status (SES), unmarried and urban patients diagnosed with low‐risk PCa. Unmarried status and low SES were associated with worse overall survival among patients undertaking AS/WW.

*Meaning*: The utilization of conservative management was affected by socioeconomic factors, which might serve as a barrier of treatment decision‐making and targeted a population in need of social support.

## 1 INTRODUCTION

Management of localized prostate cancer (PCa) remains controversial for both clinicians and patients when weighing the benefits and the potential treatment harms. Conservative management including active surveillance or watchful waiting (AS/WW) has been shown to be feasible and effective in clinically insignificant PCa with less adverse effects and limited survival disadvantages, comparing to radiotherapy or surgery (RT/Surgery).^1^, ^2^, ^3^, ^4^, ^5^ The 2021 National Comprehensive Cancer Network (NCCN) guidelines stated AS or observation (also known as WW) was recommended for very low‐, low‐, and favorable intermediate‐risk PCa based on different life expectancies.^6^ In the past 15 years, the utilization of AS/WW has significantly increased. Most recently, AS has become the dominant management for low‐risk PCa, although the absolute rates varied across different health care settings.^7^, ^8^, ^9^, ^10^

The decisions of AS/WW are often made by both clinicians and patients as resectable tumors will be reserved comparing to radical treatment. In addition, the efficacy of AS/WW depends on rigorous follow‐up, including routine prostate‐specific antigen (PSA) tests, repeated magnetic resonance imaging and prostate biopsies, etc. It highly depends on the patients' compliance.^11^, ^12^ A recent study suggested that socioeconomic status (SES) played important roles in the decision of AS/WW.^13^ SES was derived from median household income, median house value, median rent, percent below 150% of the poverty line, education index, percent working class, and percent unemployed.^14^ Besides these factors, other socioeconomic factors might be also important. For instance, in radical treatment, one of the major side effects is the reduction of sexual function,^2^ which is highly concerned based on personal marital status. Some studies suggested that the patients' compliance with AS/WW might be affected by marital status.^8^, ^9^ Another important factor would be the accessibility of medical resources. Both treatment and outcomes of PCa differed between urban and rural patients due to the different accessibility of urologists and geographic barriers.^15^, ^16^, ^17^

In order to better understand the influence of socioeconomic factors on AS/WW utilization and outcomes, we conducted this study based on the Surveillance, Epidemiology, and End Results (SEER) Prostate with Watchful Waiting Database. Our objective is to investigate the socioeconomic determinants (including marital status, residency locations, SES, etc.) of AS/WW tendency and disease survivals.

## 2 MATERIALS AND METHODS

### 2.1 Study cohort

The SEER Program collected AS/WW information for PCa cases since 2010.^18^ Using the SEER Prostate with Watchful Waiting Database, we identified 229,428 adult men diagnosed with NCCN primary localized prostate adenocarcinoma (age ≥ 18 years, one primary only, pathology‐confirmed, clinical T1‐T2, N0M0) between 2010 and 2016. The intermediate‐risk group was divided into two subgroups (favorable and unfavorable) according to the NCCN guidelines. We extracted clinical and socioeconomic variables, such as initial management (AS/WW vs RT/Surgery), age at diagnosis, year of diagnosis, PSA (prior to diagnostic biopsy of prostate and treatment), number of positive cores, race, insurance status, residency (two‐category Rural Urban Commuting Area [RUCA] codes), marital status and SES tertiles.

Two variables were transformed before analysis. The expected survival was generated from US Actuarial Life Tables (life expectancy minus age at diagnosis).^19^ The marital status included five sub‐categories: married, single, divorced, widowed, and separated. All categories other than “married” are considered as “unmarried” in subgroup analyses.

We excluded patients with NCCN high‐risk features^6^ (*n* = 38,795), missing clinical variables for risk stratification (*n* = 63,105), missing socioeconomic variables (*n* = 26,752), or patients whose initial recommended treatments were not identified or managed (*n* = 11,232, e.g., actual treatment were unknown, patients refused, or physicians decided not to treat for reasons such as the presence of comorbidities). A complete case dataset was generated based on the exclusion criteria.

The study was approved by the Institutional Review Board of Shanghai Ruijin Hospital, Shanghai, China.

### 2.2 Multiple imputations for missing data

Due to a large amount of missing data of SEER Prostate with Watchful Waiting Database,^20^ we performed multiple imputation on initial treatment (AS/WW vs RT/Surgery) using the Amelia II (version 1.8.0).^21^ Besides, both the clinical variables for risk stratification (T stage, log‐transformed PSA, biopsy Grade Group, and % of positive cores) and socioeconomic variables (race, insurance status, residency, SES, marital status) were imputed. In addition, the SEER registry was set as a cross‐sectional variable and year of diagnosis as a time‐series variable. In the present study, we only focused on the favorable‐risk PCa cases, so we classified T stage as a binary variable (T1‐T2a vs. T2b‐T2c) and set “T2NOS” as a missing value in the multiple imputation models. A multiple imputation dataset was generated under 1000 maximum resampling. All the association analysis were performed in the multiple imputation dataset repeatedly as a sensitivity analysis.

### 2.3 Statistical analysis

In descriptive statistics, continuous variables were described as median (interquartile range) and categorical variables were described as number (%). The AS/WW utilization rates between subgroups were compared using Fisher's exact test. Ordinal variables (year of diagnosis) were compared using Cochran‐Armitage tests for trend.

To identify the predictors of initial management (AS/WW vs RT/Surgery) in each risk group, we performed univariable and multivariable logistic regression and calculated crude and adjusted odds ratios (aOR), 95% confidence intervals (95% CI) and *p* values for each covariate. In SEER database, the survival months were defined as the intervals between date of diagnosis and date of last contact. The overall survival (OS) was calculated from all‐cause mortality, which means death due to any cause. The cancer‐specific survival (CSS) was calculated from deaths caused by PCa. We used Cox models to determine adjusted hazard ratios (aHR) for CSS and OS in each risk group. The multicollinearity of regression models was all checked by a variance inflation factor (VIF) and a VIF of 5 or more was considered as a multicollinearity problem.

All statistical analyses were performed with R software (version 4.1.0).^22^ A two‐sided *p* < 0.05 was considered statistically significant.

## 3 RESULTS

### 3.1 Baseline characteristics

**FIGURE 1:**
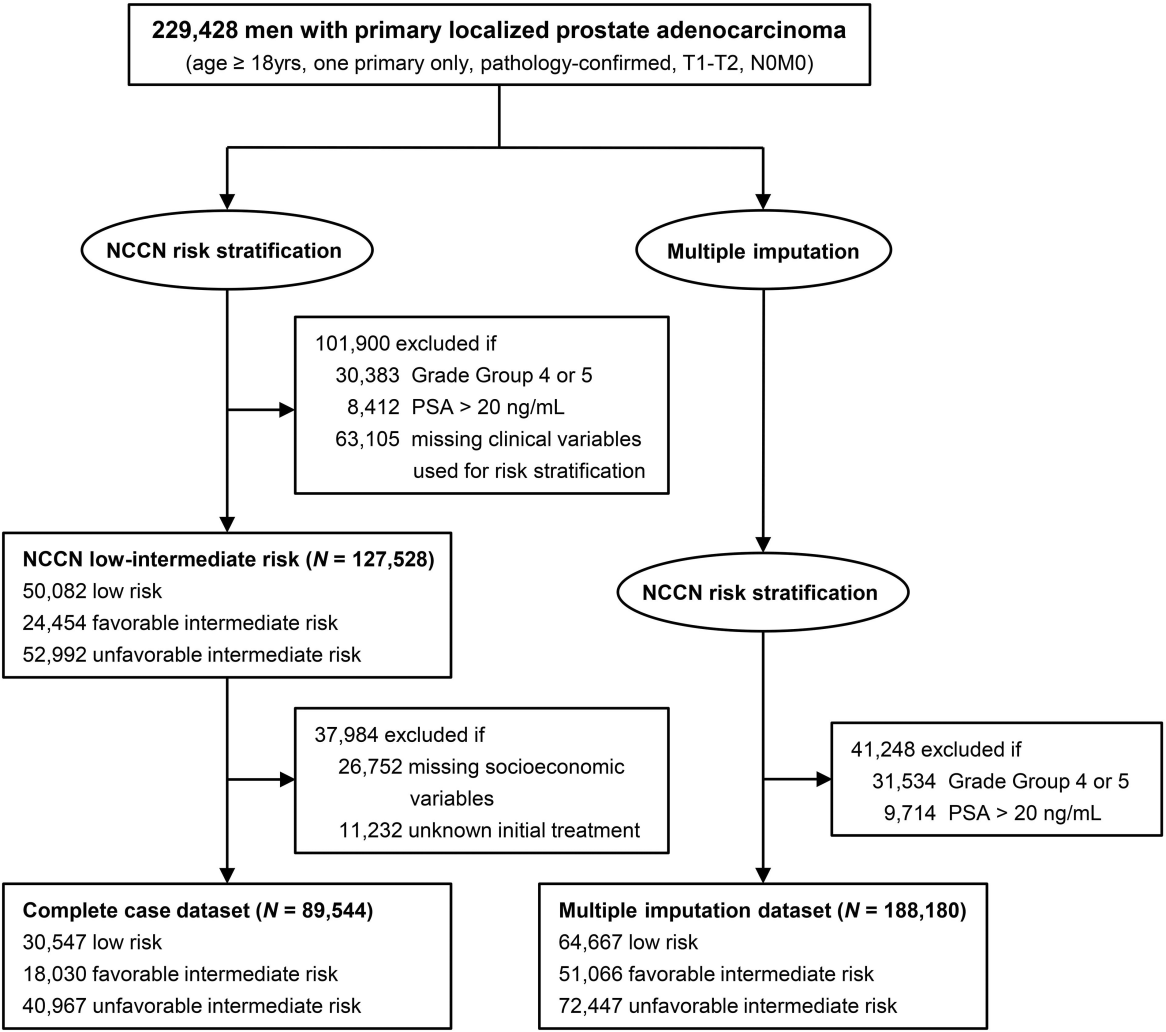
Flowchart of NCCN risk stratification and multiple imputation among men with primary localized prostate adenocarcinoma in SEER Prostate with Watchful Waiting Database. NCCN, National Comprehensive Cancer Network; PSA, prostate‐specific antigen.

The flowchart of NCCN risk stratification and multiple imputation was shown as Figure 1. The characteristics of the entire cohort, complete case dataset, and multiple imputation dataset are described in Table 1. The percentage of initial missing data ranged between 2.9% and 39.3%. As a result, only half (55.6%, *n* = 127,528) of entire cohort could be accurately classified as low‐ to intermediate‐risk PCa. On the basis of our exclusion criteria, a total of 89,544 men were included in the complete case dataset and 188,180 cases were in multiple imputation dataset (Table 1).

**TABLE 1:** Baseline characteristics of entire cohort, complete case dataset, and multiple imputation dataset in men with primary localized prostate adenocarcinoma.

Characteristic	Entire cohort (*n* = 229,428)		Complete case dataset (*n* = 89,544)	Multiple imputation dataset (*n* = 188,180)
	*N* (%)	Value	Value	Value
Age at diagnosis (years)	229,428 (100)	66 (60–72)	64 (59–69)	65 (59–70)
PSA (ng/mL)	201,801 (88.0)	6.2 (4.7–9.3)	5.8 (4.6–8.0)	5.9 (4.5–8.2)
Race				
Non‐Hispanic White	222,724 (97.1)	151,400 (68.0)	62,355 (69.6)	129,486 (68.8)
Non‐Hispanic Black		38,170 (17.1)	14,855 (16.6)	31,392 (16.7)
Hispanic		21,766 (9.8)	7761 (8.7)	18,348 (9.8)
Non‐Hispanic Others		11,388 (5.1)	4573 (5.1)	8954 (4.8)
Insurance status				
Insured	202,545 (88.3)	188,548 (93.1)	84,536 (94.4)	174,115 (92.5)
Medicaid		10,917 (5.4)	3909 (4.4)	10,580 (5.6)
Uninsured		3080 (1.5)	1099 (1.2)	3485 (1.9)
Residency				
Urban	220,438 (96.1)	204,323 (92.7)	82,952 (92.6)	174,449 (92.7)
Rural		16,115 (7.3)	6592 (7.4)	13,731 (7.3)
SES index				
Low tertile	216,591 (94.4)	59,001 (27.2)	22,633 (25.3)	49,729 (26.4)
Middle tertile		71,410 (33.0)	29,536 (33.0)	61,562 (32.7)
High tertile		86,180 (39.8)	37,375 (41.7)	76,889 (40.9)
Marital status				
Married	192,134 (83.7)	144,126 (75.0)	69,088 (77.2)	135,640 (72.1)
Single		23,738 (12.4)	10,100 (11.3)	25,014 (13.3)
Divorced		14,849 (7.7)	6827 (7.6)	16,500 (8.8)
Widowed		7583 (4.0)	2748 (3.1)	8245 (4.4)
Separated		1838 (1.0)	781 (0.9)	2781 (1.5)
T stage				
T1‐T2a		130,342 (56.8)	54,741 (61.1)	124,315 (66.1)
T2b‐T2c		65,538 (28.6)	34,803 (38.9)	63,865 (33.9)
T2NOS		33,548 (14.6)		
Biopsy Grade Group				
1	222,254 (96.9)	105,186 (47.3)	45,538 (50.9)	105,123 (55.9)
2		60,651 (27.3)	30,101 (33.6)	58,790 (31.2)
3		26,034 (11.7)	13,905 (15.5)	24,267 (12.9)
4		18,766 (8.4)		
5		11,617 (5.2)		
% of positive cores	139,169 (60.7)	0.29 (0.16–0.50)	0.25 (0.15–0.48)	0.26 (0.13–0.46)
NCCN risk group				
Low	127,528 (55.6)	50,082 (39.3)	30,547 (34.1)	64,667 (34.4)
Favorable intermediate		24,454 (19.2)	18,030 (20.1)	51,066 (27.1)
Unfavorable intermediate		52,992 (41.6)	40,967 (45.8)	72,447 (38.5)
Initial management				
RT/Surgery	185,075 (80.7)	159,692 (86.3)	74,730 (83.5)	154,015 (81.8)
AS/WW		25,383 (13.7)	14,814 (16.5)	34,165 (18.2)
Follow‐up duration, months	229,428 (100)	41 (18–63)	42 (19–63)	43 (20–64)
Overall mortality	229,428 (100)	11,857 (5.2)	2415 (2.7)	7030 (3.7)
Cancer‐specific mortality	229,428 (100)	2615 (1.1)	247 (0.3)	757 (0.4)

*Note*: Continuous variables were described as median (interquartile range) and categorical variables were described as number (percentage).

Abbreviation: AS/WW, Active surveillance/watchful waiting; NCCN, National Comprehensive Cancer Network; PSA, prostate‐specific antigen; SES, socioeconomic status.

### 3.2 Socioeconomic factors and AS/WW trends

**FIGURE 2:**
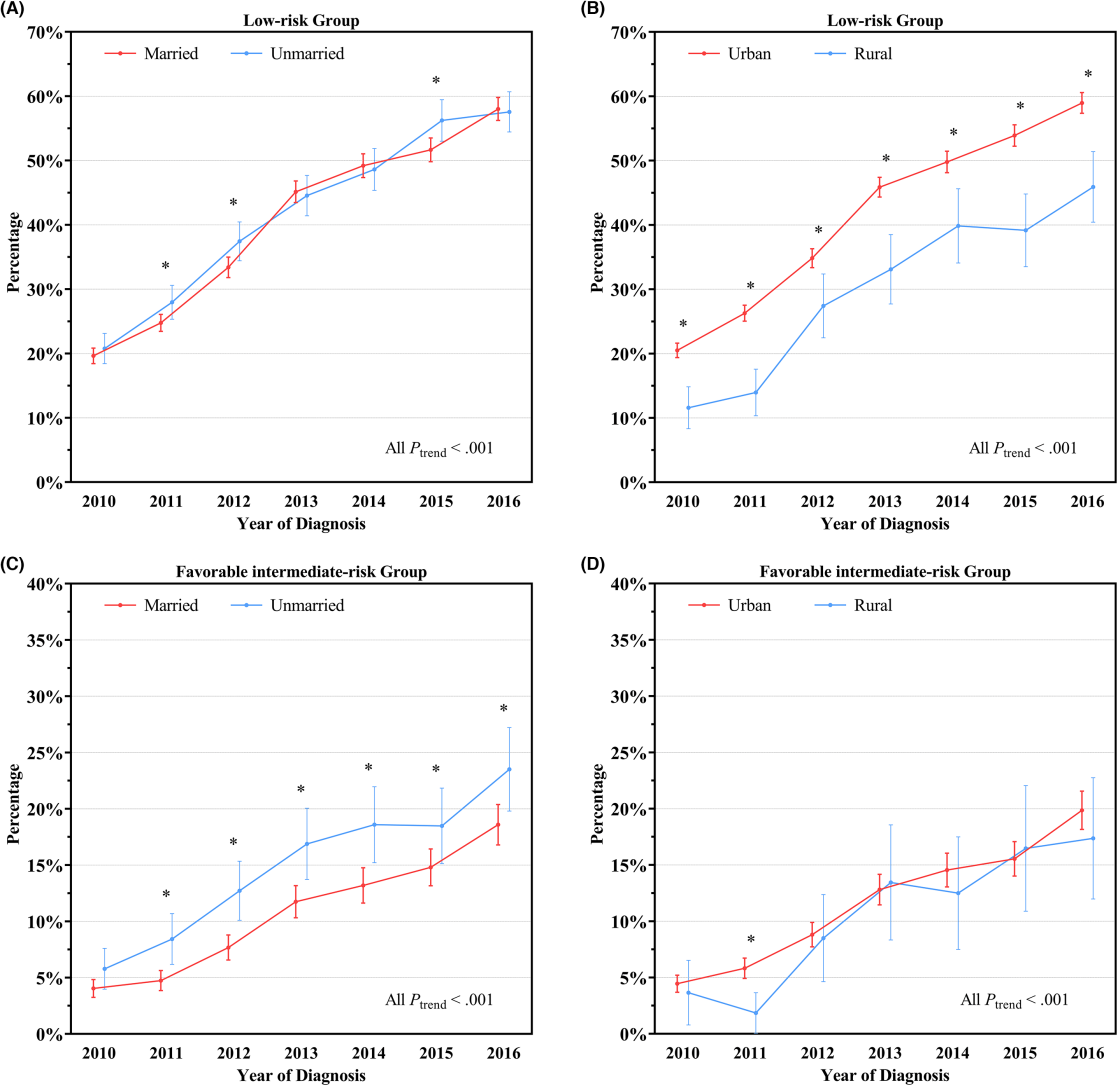
Yearly rates of active surveillance/watchful waiting management among men with NCCN favorable‐risk prostate cancer from 2010 to 2016 in complete case dataset. NCCN, National Comprehensive Cancer Network. Results were shown by marital status (married vs unmarried), residency (urban vs rural) and NCCN risk stratification (low‐ and favorable intermediate‐risk groups). Error bars represent 95% confidence intervals. The percentages between subgroups were compared using Fisher's exact test (an asterisk represented a significant difference in a specific year). The yearly trends were assessed using Cochran–Armitage test (*p*_trend_).

The AS/WW rates increased over time regardless of marital status and residency in NCCN low‐ and favorable intermediate‐risk groups from 2010 to 2016 (all *p*_trend_ <0.001, Figure 2). In low‐risk group, AS/WW rates were significantly lower in married patients than in unmarried patients (38.3% vs 40.8%, *p <* 0.001), although significant difference were only found in 2011 and 2012 in subgroups analyses by year of diagnosis (Figure 2). Notably, urban patients had significantly higher AS/WW rates than rural patients within each year (all *p <* 0.001). In favorable intermediate‐risk group, similarly, AS/WW rates were significantly lower in married patients than in unmarried patients (10.2% vs 14.4%, *p <* 0.001), and the difference were still significant except for 2010 in each year. While no significant difference was observed between urban and rural patients (*p* = 0.38). Similar results were found in multiple imputation dataset (Figure S1).

Among patients with low‐risk PCa (*n* = 30,547), multivariable logistic regression analysis showed that unmarried patients had a significant increased AS/WW rates compared to married patients (aOR, 1.20 [95%CI, 1.12–1.28]; *p* < 0.001). Similar results were found in sub‐categories except for “widowed” (aOR_single_, 1.25 [95% CI, 1.14–1.36], *p <* 0.001; aOR_divorced_, 1.23 [95% CI, 1.10–1.36], *p <* 0.001; aOR_separated_, 1.47 [95% CI, 1.08–1.99], *p* = 0.01, Table 2). Rural patients had ~0.77‐fold lower likelihood of AS/WW management than urban patients (95% CI, 0.68–0.87, *p <* 0.001). In addition, non‐Hispanic Black and Medicaid beneficiaries were considered having less preference of AS/WW management than others (all *p <* 0.05). Patients in middle and high SES tertile would prefer AS/WW than low group (aOR_middle_: 1.29, aOR_high_: 1.66, all *p <* 0.001). Similar results were found in multiple imputation dataset (Table S1).

**TABLE 2:** Effect of baseline and socioeconomic factors on initial management (AS/WW versus RT/Surgery) among men with NCCN favorable‐risk prostate cancer in complete case dataset.

Characteristic	Low risk (*n* = 30,547)				Favorable‐intermediate risk (*n* = 18,030)			
	Crude OR (95% CI)	*p*	Adjusted OR (95% CI)	*p*	Crude OR (95% CI)	*p*	Adjusted OR (95% CI)	*p*
Expected survival (years)								
≥ 10	1.0 (Ref.)	<0.001	1.0 (Ref.)	0.001	1.0 (Ref.)	<0.001	1.0 (Ref.)	<0.001
< 10	0.74 (0.66–0.82)		1.28 (1.11–1.47)		2.89 (2.45–3.40)		2.36 (1.97–2.84)	
Year of diagnosis	1.34 (1.32–1.35)	<0.001	1.40 (1.38–1.42)	<0.001	1.30 (1.27–1.34)	<0.001	1.32 (1.28–1.35)	<0.001
PSA (ng/ml)	1.40 (1.33–1.46)	<0.001	1.09 (1.02–1.16)	0.007	6.58 (5.89–7.36)	<0.001	5.28 (4.70–5.92)	<0.001
No. positive cores								
< 3	1.0 (Ref.)	<0.001	1.0 (Ref.)	<0.001	1.0 (Ref.)	<0.001	1.0 (Ref.)	<0.001
≥ 3	0.43 (0.41–0.46)		0.39 (0.36–0.41)		0.42 (0.38–0.46)		0.43 (0.38–0.47)	
Race								
Non‐Hispanic White	1.0 (Ref.)		1.0 (Ref.)		1.0 (Ref.)		1.0 (Ref.)	
Non‐Hispanic Black	0.82 (0.77–0.88)	<0.001	0.91 (0.84–0.99)	0.025	1.05 (0.93–1.20)	0.416	1.02 (0.88–1.18)	0.823
Hispanic	0.89 (0.82–0.96)	0.004	0.92 (0.83–1.02)	0.100	1.00 (0.84–1.19)	0.996	0.91 (0.75–1.10)	0.316
Others	1.25 (1.13–1.39)	<0.001	1.06 (0.94–1.20)	0.353	1.40 (1.15–1.70)	0.001	1.00 (0.81–1.24)	0.995
Insurance status								
Insured	1.0 (Ref.)		1.0 (Ref.)		1.0 (Ref.)		1.0 (Ref.)	
Medicaid	0.74 (0.65–0.83)	<0.001	0.83 (0.71–0.96)	0.011	1.57 (1.27–1.95)	<0.001	1.15 (0.90–1.46)	0.264
Uninsured	1.36 (1.10–1.69)	0.004	1.53 (1.17–1.99)	0.002	1.33 (0.89–1.99)	0.161	1.08 (0.70–1.66)	0.739
Residency								
Urban	1.0 (Ref.)		1.0 (Ref.)		1.0 (Ref.)		1.0 (Ref.)	
Rural	0.63 (0.57–0.69)	<0.001	0.77 (0.68–0.87)	<0.001	0.92 (0.76–1.10)	0.355	0.90 (0.73–1.11)	0.308
Marital status								
Married	1.0 (Ref.)		1.0 (Ref.)		1.0 (Ref.)		1.0 (Ref.)	
Single	1.19 (1.11–1.28)	<0.001	1.25 (1.14–1.36)	<0.001	1.43 (1.24–1.64)	<0.001	1.37 (1.18–1.60)	<0.001
Divorced	1.14 (1.05–1.25)	0.002	1.23 (1.10–1.36)	<0.001	1.47 (1.25–1.73)	<0.001	1.43 (1.19–1.71)	<0.001
Widowed	0.77 (0.67–0.88)	<0.001	0.90 (0.75–1.07)	0.214	1.54 (1.19–1.99)	0.001	1.22 (0.92–1.62)	0.165
Separated	1.30 (1.01–1.68)	0.042	1.47 (1.08–1.99)	0.014	2.29 (1.55–3.40)	<0.001	2.84 (1.83–4.40)	<0.001
SES status								
Low tertile	1.0 (Ref.)		1.0 (Ref.)		1.0 (Ref.)		1.0 (Ref.)	
Middle tertile	1.33 (1.25–1.42)	<0.001	1.29 (1.19–1.39)	<0.001	1.09 (0.96–1.24)	0.166	1.28 (1.11–1.47)	0.001
High tertile	1.71 (1.62–1.82)	<0.001	1.66 (1.53–1.79)	<0.001	1.15 (1.02–1.30)	0.020	1.42 (1.23–1.63)	<0.001

Abbreviation: AS/WW, active surveillance/watchful waiting; NCCN, National Comprehensive Cancer Network; OR, odds ratio; PSA, prostate‐specific antigen; Ref, reference; RT, radiation therapy; SES, socioeconomic status.

Among patients in favorable intermediate‐risk group (*n* = 18,030), unmarried patients tended to receive AS/WW compared to married patients (aOR, 1.41 [95%CI, 1.26–1.59]; *p* < 0.001). Similar results were also found in sub‐categories except for “widowed” (*p* < 0.001, Table 2). Higher SES was associated with higher rates of AS/WW (aOR_middle_: 1.28, aOR_high_: 1.42, all *p <* 0.001). However, no statistical difference was found among different races, insurance statuses and residency. All the VIFs of covariables were <2.0 in collinearity analyses, which indicated that no multicollinearity problems were found in our logistic regression models. Similar results were found in multiple imputation dataset (Table S1).

### 3.3 Socioeconomic factors and survival outcomes

The median follow‐up was 41 months (range: 18–63 months) in entire cohort. Among men with favorable‐risk PCa (*n* = 48,577), 13,875 (28.6%) men received AS/WW as initial management (Table 3). We then performed survival analysis in the AS/WW subset. Interestingly, men in high SES tertile had better outcomes than low tertile group (aHR, 0.08 [95% CI, 0.01–0.69]; *p* = 0.02). Neither the NCCN risk group nor the other socioeconomic factors (race, insurance status, and residency) were not significantly associated with CSS. A significantly worse OS was observed among unmarried patients comparing to married group (aHR, 1.98 [95%CI, 1.50–2.60]; *p* < 0.001). However, “single” and “divorced” patients had significantly worse OS than married patients (aHR_single_, 1.88 [95% CI, 1.29–2.73], *p* = 0.001; aHR_divorced_, 2.47 [95% CI, 1.72–3.55], *p* < 0.001, Table 3). Repeated subgroup analyses showed similar results in multiple imputation dataset (Table S2).

**TABLE 3:** Multivariable adjusted hazard ratios of socioeconomic factors on cancer‐specific survival and overall survival among favorable‐risk patients receiving AS/WW in complete case dataset.

Characteristic	No. of total patients	Cancer‐specific survival		Overall survival	
		Adjusted HR (95% CI)	*p*	Adjusted HR (95% CI)	*p*
Age at diagnosis (years)	13,875	1.05 (0.98–1.13)	0.183	1.09 (1.07–1.11)	<0.001
Year of diagnosis		0.99 (0.60–1.65)	0.980	1.01 (0.91–1.12)	0.819
PSA (ng/ml)		3.25 (0.62–17.09)	0.163	1.15 (0.85–1.55)	0.356
No. positive cores					
< 3	9290	1.0 (Ref.)	0.211	1.0 (Ref.)	0.591
≥ 3	3078	2.06 (0.66–6.41)		1.08 (0.81–1.46)	
Race					
Non‐Hispanic White	9869	1.0 (Ref.)		1.0 (Ref.)	
Non‐Hispanic Black	2009	2.19 (0.55–8.75)	0.267	0.90 (0.62–1.30)	0.567
Hispanic	1179	2.44 (0.47–12.72)	0.290	0.80 (0.48–1.36)	0.415
Others	818	3.07 (0.35–26.91)	0.311	0.44 (0.18–1.08)	0.075
Insurance status					
Insured	13,158	1.0 (Ref.)		1.0 (Ref.)	
Medicaid	529	—	—	0.64 (0.28–1.47)	0.293
Uninsured	188	—	—	0.73 (0.18–2.95)	0.657
Residency					
Rural	13,101	1.0 (Ref.)	0.309	1.0 (Ref.)	0.671
Urban	774	2.30 (0.46–11.41)		1.11 (0.69–1.79)	
Marital status					
Married	10,459	1.0 (Ref.)		1.0 (Ref.)	
Single	1752	2.09 (0.53–8.29)	0.293	1.88 (1.29–2.73)	0.001
Divorced	1150	1.47 (0.30–7.28)	0.636	2.47 (1.72–3.55)	<0.001
Widowed	373	—	—	1.38 (0.76–2.52)	0.296
Separated	141	—	—	1.47 (0.46–4.65)	0.516
SES status					
Low tertile	2885	1.0 (Ref.)		1.0 (Ref.)	
Middle tertile	4398	0.53 (0.16–1.78)	0.304	0.62 (0.45–0.85)	0.003
High tertile	6592	0.08 (0.01–0.69)	0.022	0.33 (0.23–0.47)	<0.001
Risk group					
Low	11,873	1.0 (Ref.)		1.0 (Ref.)	
Favorable‐intermediate	2002	1.22 (0.28–5.31)	0.793	1.57 (1.13–2.17)	0.006

Abbreviations: AS/WW, active surveillance/watchful waiting; HR, hazard ratio; PSA, prostate‐specific antigen; NCCN, National Comprehensive Cancer Network; Ref, reference; RT, radiation therapy; SES, socioeconomic status.

## 4 DISCUSSION

The current study presented a comprehensive analysis of the association between socioeconomic factors and AS/WW utilization, as well as the disease outcomes. To the best of our knowledge, this the first study presents the association between marital status, residency and AS/WW based on the US population. We found that (1) the AS/WW rates significantly increased over time from 2010 to 2016. Such trends remained significant when stratified by clinical risk, marital status or residency; (2) Unmarried patients (single, divorced and separated, except for widowed) had significantly higher AS/WW rates than married patients in both low‐ and favorable intermediate‐risk groups; (3) Rural patients had significantly lower likelihood (~0.77‐fold) of AS/WW use than urban patients only in the low‐risk group; (4) Unmarried patients had significantly worse OS than married patients after receiving AS/WW.

Several retrospective studies demonstrated that the AS/WW utilization rates for low‐ to intermediate‐risk PCa have increased significantly since 2010 in different US population‐based cohorts.^7^, ^9^, ^10^, ^23^ We found that the yearly trends were still significant after being stratified by marital status and residency, which indicated the preference for AS/WW was rising nationwide.

We found unmarried status and its sub‐categories (except for “widowed”) were significantly associated with higher rates of AS/WW within each clinical risk group. The difference might be due to that widowed status was not an active, voluntary choice, comparing to other unmarried components (single, divorced, or separated). Remarkably, in the low‐risk group, the unmarried patients had ~1.20‐fold higher likelihood of AS/WW management than married patients (95% CI: 1.12–1.28), which was similar as the results based on US Veterans database from 2005 to 2015 (aOR: 1.18, 95% CI: 1.15–1.21).^9^ Similar conclusions were also reported in Sweden population.^8^ A potential explanation is that the unmarried patients are likely to pay more attention to the quality of life and receive a newer form of treatment.

Mahal et al and Butler et al reported that several factors (including age, year of diagnosis, and Yost index) were predictive of AS/WW utilization in the SEER database, which were consistent with our results based on an extra follow‐up year (2010–2016).^23^, ^24^ Another study based on SEER database demonstrated that African American men would be more likely to undergo AS/WW than other races in low‐risk PCa patients.^25^ Similar results were reported in the intermediate‐risk group by Butler et al.^23^ However, in the present study, when analyses were limited in non‐Hispanic population, African American men were less likely to undergo AS/WW than Caucasian men in the low‐risk group in our adjusted model. In addition, no significant association between AS/WW rates and ethnicities (non‐Hispanic white or black patients) were observed in the favorable intermediate‐risk group. The definition of Hispanic/Latino population varies by regions in United States. Therefore, the effect of race on survival can be more precise when limited in non‐Hispanic population, due to the complexity of the Hispanic population.

A recent study by Maganty et al indicated that rural PCa patients were less likely to undergo any treatment in entire cohort and different risk groups.^15^ In our study, we found that rural and low‐SES patients were less likely to undergo AS/WW, and a significant difference was found in the low‐risk group. As AS/WW requires regular follow‐up, multiple PSA tests and annual prostate biopsy, the relatively low accessibility of medical resources (such as low urologist density, long travel distance to the clinic) would be a major concern for rural or low‐SES patients. In addition, rural residency is usually associated with relatively low household income. It would be another factor that could have driven the patients from choosing AS/WW, as studies showed that despite the cost‐effective of AS/WW at the population level, AS/WW would increase costs on disease management at individual level.^26^, ^27^

In the perspective of clinical practice, AS/WW, as a recommended management by NCCN for low‐ and favorable intermediate‐risk PCa, would not lead to worse CSS. Such results were confirmed in our current study and other reported studies.^23^, ^24^ Both married status and high SES tertile served as protective factors for men receiving AS/WW, even after adjusting for NCCN risk group.

Missing data are an unavoidable issue in all medical studies, especially for the analyses based on the population‐based databases.^28^, ^29^ Jeong et al reported the new SEER Prostate with Watchful Waiting Database contained a large amount of missing data, including the necessary variables for NCCN risk classification.^20^ As a result, simply excluding cases with missing data will bring potential bias. In the present study, the multiple imputation dataset (*N* = 188,180) is approximately 2.1 times larger than the complete case dataset (*N* = 89,544, Figure 1), which is similar to the previous study (118,821 vs. 257,060, ~2.2 times).^20^ Furthermore, all the regression analyses were repeated in the multiple imputation dataset, which strengthened the original results and conclusions and reduced the bias. Recently, the multiple imputation has been widely applied in clinical studies.^30^, ^31^ As for an imputation approach, it is important to assess whether data were missing at random (MAR).^28^ In other words, the multiple imputation process is implemented based on the MAR assumption.^32^ Missing completely at random (MCAR) is the optimal but rare pattern in clinical studies. More importantly, we can consider MAR as an approximation of MCAR when including as many covariates as possible in the models of the current study.^33^

Several limitations of this study should be noted. First, we did not perform analyses in very low‐risk PCa patients due to the lack of data (cancer percentage in each positive core, PSA density, or volume^6^, ^18^). However, the benefits of AS/WW for patients with very low‐risk PCa may overcome the harms compared to radical treatments. It is less important to perform a similar evaluation in these patients. Further analyses are optional if additional data can be acquired. Second, AS involves active monitoring and potential curative treatment after progression, and WW means monitoring and palliative therapy if needed. The dataset did not distinguish AS from WW, which may reduce the generalizability of our results to either AS or WW. It is worth further investigation. Third, the median follow‐up was less than 4 years, and longer observation will be needed. The time‐dependent variables (such as marital status, income, and residency) were collected at a single time of diagnosis, so the timing of assessment cannot be evaluated in the present study. Fourth, large numbers of cases lacked necessary characteristics for the NCCN risk stratification.^20^ However, we performed sensitivity analysis in multiple imputation dataset and showed similar results as complete case dataset. Our conclusions from this retrospective cohort still need high‐level evidence or models for further verification.

### 4.1 Conclusion

In conclusion, marital status, residency and SES (including factors of income, education and employment) independently influenced the utilization of AS/WW and served as a barrier to treatment decision‐making among favorable‐risk PCa patients. Married and high SES patients undertaking AS/WW would have better OS. Personalized social supports should be provided to these patients to improve the disease outcome.

## AUTHOR CONTRIBUTIONS

**Da Huang:** Data curation (equal); formal analysis (equal); funding acquisition (equal); methodology (equal); visualization (equal); writing – original draft (equal). **Xiaohao Ruan:** Data curation (equal); formal analysis (equal); visualization (equal); writing – original draft (equal). **Jingyi Huang:** Data curation (equal); formal analysis (equal); investigation (equal); writing – original draft (equal). **Ning Zhang:** Resources (equal); software (equal). **Guangliang Jiang:** Resources (equal); software (equal). **Yi Gao:** Resources (equal); software (equal). **Danfeng Xu:** Conceptualization (equal); supervision (equal); writing – review and editing (equal). **Rong Na:** Conceptualization (equal); funding acquisition (equal); supervision (equal); writing – review and editing (equal).

## FUNDING INFORMATION

This work was in supported by grants from National Natural Science Foundation of China (grant nos 81772741, 1972405, 81972645, and 82173045), Shanghai Youth Talent Support Program, and the Shanghai Rising‐Star Incubator Program (22YF1440500). All the funders had no role in study design, data collection, data analysis, interpretation, and writing of the report.

## CONFLICT OF INTEREST STATEMENT

The authors declare that they have no competing interests.

## Supporting information


Appendix S1.

## Data Availability

We thanked SEER program to approve our protocol and provide the custom datasets. All data used in this research are publicly available to qualified researchers on application to the SEER program (http://seer.cancer.gov/).
