# Supplementary material for: Socioeconomic determinants are associated with the utilization and outcomes of active surveillance or watchful waiting in favorable‐risk prostate cancer
Source: Cancer Med. 2023 Feb 2;12(8):9868–78. doi: 10.1002/cam4.5650 (PMC10166939; doi:10.1002/cam4.5650)
Supplement: Supplementary file 1 — Appendix S1. [file CAM4-12-9868-s001.docx]

**Supplementary Material**

1. **Figure S1.** Yearly rates of active surveillance/watchful waiting management among men with NCCN favorable-risk prostate cancer from 2010 to 2016 in multiple imputation dataset.
2. **Table S1**. Effect of baseline and socioeconomic factors on initial management (AS/WW versus RT/Surgery) among men with NCCN favorable-risk prostate cancer in multiple imputation dataset.
3. **Table S2.** Multivariable adjusted hazard ratios of socioeconomic factors on cancer-specific survival and overall survival among favorable-risk patients receiving AS/WW in multiple imputation dataset.
4. **Table S3.** Effect of baseline and socioeconomic factors on initial management (AS/WW versus RT/Surgery) among men with NCCN unfavorable-risk prostate cancer in complete case dataset and multiple imputation dataset.

**Figure S1. Yearly rates of active surveillance/watchful waiting management among men with NCCN favorable-risk prostate cancer from 2010 to 2016 in multiple imputation dataset.**


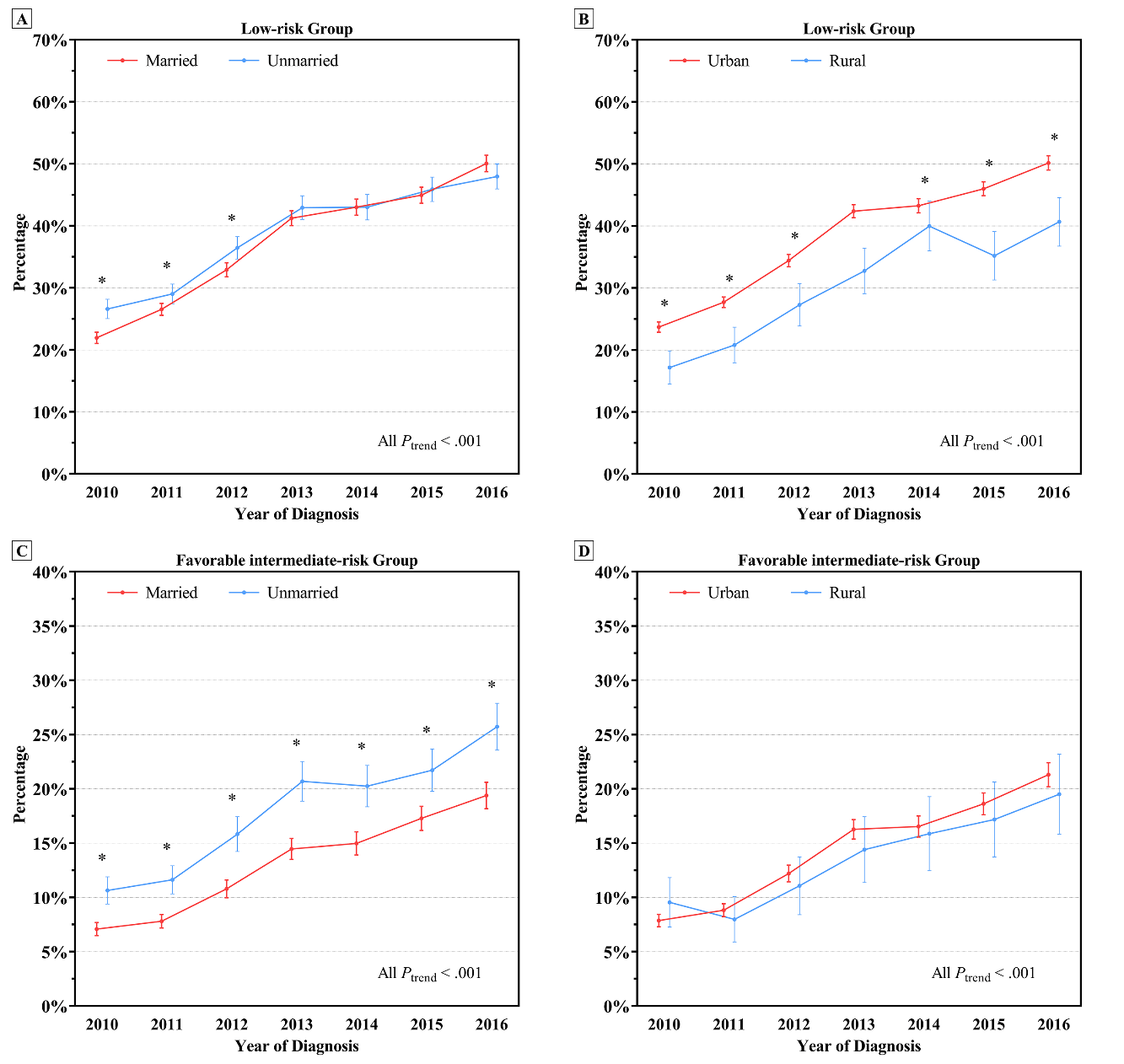


Abbreviation: NCCN, National Comprehensive Cancer Network.

Results were shown by marital status (married vs unmarried), residency (urban vs rural) and NCCN risk stratification (low- and favorable intermediate-risk groups). Error bars represent 95% confidence intervals. The percentages between subgroups were compared using Fisher’s exact test (an asterisk represented a significant difference in a specific year). The yearly trends were assessed using Cochran–Armitage test (*P*_trend_).

**Table S1. Effect of baseline and socioeconomic factors on initial management (AS/WW versus RT/Surgery) among men with NCCN favorable-risk prostate cancer in multiple imputation dataset.**

| Characteristic | Low risk (n = 64,667) | | | | Favorable-intermediate risk (n = 51,066) | | | |
| --- | --- | --- | --- | --- | --- | --- | --- | --- |
|  | Crude OR (95% CI) | *P* | Adjusted OR (95% CI) | *P* | Crude OR (95% CI) | *P* | Adjusted OR (95% CI) | *P* |
| Expected survival |  |  |  |  |  |  |  |  |
| ≥10 yrs | 1.0 (Ref.) |  | 1.0 (Ref.) |  | 1.0 (Ref.) |  | 1.0 (Ref.) |  |
| <10 yrs | 0.70 (0.65-0.74) | <0.001 | 0.77 (0.71-0.82) | <0.001 | 2.08 (1.92-2.26) | <0.001 | 1.78 (1.63-1.94) | <0.001 |
| Year of diagnosis | 1.22 (1.21-1.23) | <0.001 | 1.23 (1.22-1.24) | <0.001 | 1.21 (1.20-1.23) | <0.001 | 1.21 (1.20-1.23) | <0.001 |
| PSA, ng/mL | 1.18 (1.14-1.22) | <0.001 | 1.24 (1.20-1.29) | <0.001 | 3.04 (2.88-3.20) | <0.001 | 2.62 (2.49-2.77) | <0.001 |
| No. positive cores |  |  |  |  |  |  |  |  |
| <3 | 1.0 (Ref.) |  | 1.0 (Ref.) |  | 1.0 (Ref.) |  | 1.0 (Ref.) |  |
| ≥3 | 0.50 (0.48-0.52) | <0.001 | 0.49 (0.47-0.50) | <0.001 | 0.51 (0.49-0.54) | <0.001 | 0.51 (0.48-0.54) | <0.001 |
| Race |  |  |  |  |  |  |  |  |
| Non-Hispanic White | 1.0 (Ref.) |  | 1.0 (Ref.) |  | 1.0 (Ref.) |  | 1.0 (Ref.) |  |
| Non-Hispanic Black | 0.83 (0.79-0.87) | <0.001 | 0.90 (0.86-0.95) | <0.001 | 0.97 (0.90-1.04) | 0.417 | 0.93 (0.86-1.00) | 0.063 |
| Hispanic | 0.84 (0.80-0.89) | <0.001 | 0.87 (0.82-0.92) | <0.001 | 0.97 (0.89-1.05) | 0.444 | 0.89 (0.81-0.98) | 0.017 |
| Others | 1.22 (1.13-1.31) | <0.001 | 1.14 (1.05-1.23) | 0.001 | 1.26 (1.13-1.42) | <0.001 | 1.02 (0.91-1.15) | 0.731 |
| Insurance status |  |  |  |  |  |  |  |  |
| Insured | 1.0 (Ref.) |  | 1.0 (Ref.) |  | 1.0 (Ref.) |  | 1.0 (Ref.) |  |
| Medicaid | 0.72 (0.67-0.77) | <0.001 | 0.72 (0.67-0.78) | <0.001 | 1.25 (1.12-1.39) | <0.001 | 0.98 (0.88-1.11) | 0.790 |
| Uninsured | 1.11 (0.98-1.24) | 0.093 | 1.11 (0.99-1.26) | 0.081 | 1.48 (1.25-1.76) | <0.001 | 1.39 (1.15-1.66) | <0.001 |
| Residency |  |  |  |  |  |  |  |  |
| Urban | 1.0 (Ref.) |  | 1.0 (Ref.) |  | 1.0 (Ref.) |  | 1.0 (Ref.) |  |
| Rural | 0.72 (0.67-0.77) | <0.001 | 0.82 (0.77-0.88) | <0.001 | 0.95 (0.86-1.05) | 0.354 | 0.96 (0.86-1.07) | 0.439 |
| Marital status |  |  |  |  |  |  |  |  |
| Married | 1.0 (Ref.) |  | 1.0 (Ref.) |  | 1.0 (Ref.) |  | 1.0 (Ref.) |  |
| Single | 1.14 (1.08-1.19) | <0.001 | 1.20 (1.14-1.26) | <0.001 | 1.40 (1.30-1.50) | <0.001 | 1.37 (1.27-1.48) | <0.001 |
| Divorced | 1.13 (1.07-1.19) | <0.001 | 1.21 (1.14-1.28) | <0.001 | 1.60 (1.47-1.74) | <0.001 | 1.57 (1.43-1.71) | <0.001 |
| Widowed | 0.91 (0.84-0.98) | 0.019 | 1.00 (0.92-1.09) | 0.948 | 1.59 (1.42-1.79) | <0.001 | 1.32 (1.17-1.50) | <0.001 |
| Separated | 1.17 (1.03-1.33) | 0.016 | 1.28 (1.12-1.47) | <0.001 | 1.70 (1.41-2.05) | <0.001 | 1.68 (1.38-2.04) | <0.001 |
| SES status |  |  |  |  |  |  |  |  |
| Low tertile | 1.0 (Ref.) |  | 1.0 (Ref.) |  | 1.0 (Ref.) |  | 1.0 (Ref.) |  |
| Middle tertile | 1.28 (1.23-1.34) | <0.001 | 1.25 (1.20-1.31) | <0.001 | 1.17 (1.09-1.25) | <0.001 | 1.26 (1.17-1.36) | <0.001 |
| High tertile | 1.56 (1.50-1.62) | <0.001 | 1.52 (1.45-1.59) | <0.001 | 1.16 (1.09-1.24) | <0.001 | 1.34 (1.24-1.44) | <0.001 |

Abbreviation: AS/WW, active surveillance/watchful waiting; RT, radiation therapy; NCCN, National Comprehensive Cancer Network; OR, odds ratio; PSA, prostate-specific antigen; Ref, reference; SES, socioeconomic status.

**Table S2. Multivariable adjusted hazard ratios of socioeconomic factors on cancer-specific survival and overall survival among favorable-risk patients receiving AS/WW in multiple imputation dataset.**

| Characteristic | No. of total patients | Cancer-specific survival | | Overall survival | |
| --- | --- | --- | --- | --- | --- |
|  |  | Adjusted HR (95% CI) | *P* | Adjusted HR (95% CI) | *P* |
| Age at diagnosis, yrs | 30,490 | 1.09 (1.06-1.13) | <0.001 | 1.10 (1.09-1.10) | <0.001 |
| Year of diagnosis |  | 0.97 (0.79-1.19) | 0.783 | 0.96 (0.92-1.02) | 0.172 |
| PSA, ng/mL |  | 1.20 (0.73-1.96) | 0.476 | 0.99 (0.87-1.12) | 0.825 |
| No. positive cores |  |  |  |  |  |
| <3 | 22,294 | 1.0 (Ref.) |  | 1.0 (Ref.) |  |
| ≥3 | 8,196 | 2.02 (1.24-3.28) | 0.005 | 1.11 (0.96-1.28) | 0.173 |
| Race |  |  |  |  |  |
| Non-Hispanic White | 21,569 | 1.0 (Ref.) |  | 1.0 (Ref.) |  |
| Non-Hispanic Black | 4,404 | 1.04 (0.49-2.21) | 0.915 | 1.06 (0.88-1.29) | 0.528 |
| Hispanic | 2,866 | 1.57 (0.73-3.36) | 0.250 | 0.63 (0.47-0.83) | 0.001 |
| Others | 1,651 | 1.24 (0.44-3.48) | 0.681 | 0.53 (0.35-0.79) | 0.002 |
| Insurance status |  |  |  |  |  |
| Insured | 28,372 | 1.0 (Ref.) |  | 1.0 (Ref.) |  |
| Medicaid | 1,487 | 0.82 (0.25-2.66) | 0.744 | 1.59 (1.22-2.06) | 0.001 |
| Uninsured | 631 | / | / | 1.13 (0.72-1.79) | 0.589 |
| Residency |  |  |  |  |  |
| Rural | 28,657 | 1.0 (Ref.) |  | 1.0 (Ref.) |  |
| Urban | 1,833 | 1.21 (0.50-2.91) | 0.668 | 1.07 (0.84-1.37) | 0.557 |
| Marital status |  |  |  |  |  |
| Married | 21,067 | 1.0 (Ref.) |  | 1.0 (Ref.) |  |
| Single | 4,494 | 1.51 (0.75-3.06) | 0.248 | 1.60 (1.33-1.92) | <0.001 |
| Divorced | 3,039 | 1.83 (0.91-3.69) | 0.092 | 1.70 (1.40-2.07) | <0.001 |
| Widowed | 1,356 | 1.65 (0.72-3.76) | 0.234 | 1.35 (1.05-1.73) | 0.018 |
| Separated | 534 | 2.06 (0.49-8.58) | 0.321 | 1.81 (1.19-2.75) | 0.006 |
| SES status |  |  |  |  |  |
| Low tertile | 6,672 | 1.0 (Ref.) |  | 1.0 (Ref.) |  |
| Middle tertile | 9,830 | 0.67 (0.35-1.26) | 0.211 | 0.69 (0.59-0.81) | <0.001 |
| High tertile | 13,988 | 0.70 (0.37-1.32) | 0.277 | 0.47 (0.39-0.56) | <0.001 |
| Risk group |  |  |  |  |  |
| Low | 23,553 | 1.0 (Ref.) |  | 1.0 (Ref.) |  |
| Favorable-intermediate | 6,937 | 1.79 (1.05-3.04) | 0.032 | 1.41 (1.21-1.64) | <0.001 |

Abbreviation: NCCN, National Comprehensive Cancer Network; HR, hazard ratio; PSA, prostate-specific antigen; Ref, reference; SES, socioeconomic status; RT, radiation therapy; AS/WW, active surveillance/watchful waiting.

**Table S3. Effect of baseline and socioeconomic factors on initial management (AS/WW versus RT/Surgery) among men with NCCN unfavorable-risk prostate cancer in complete case dataset and multiple imputation dataset.**

| Characteristic | Unfavorable-intermediate risk (n = 40,967) | | | | Unfavorable-intermediate risk in multiple imputation (n = 72,477) | | | |
| --- | --- | --- | --- | --- | --- | --- | --- | --- |
|  | Crude OR (95% CI) | *P* | Adjusted OR (95% CI) | *P* | Crude OR  (95% CI) | *P* | Adjusted OR  (95% CI) | *P* |
| Expected survival |  |  |  |  |  |  |  |  |
| ≥10 yrs | 1.0 (Ref.) |  | 1.0 (Ref.) |  | 1.0 (Ref.) |  | 1.0 (Ref.) |  |
| <10 yrs | 5.03 (4.31-5.88) | <0.001 | 4.76 (3.99-5.68) | <0.001 | 2.99 (2.76-3.25) | <0.001 | 2.74 (2.51-2.99) | <0.001 |
| Year of diagnosis | 1.15 (1.11-1.19) | <0.001 | 1.16 (1.12-1.20) | <0.001 | 1.08 (1.06-1.10) | <0.001 | 1.09 (1.07-1.10) | <0.001 |
| PSA, ng/mL | 0.88 (0.74-1.01) | <0.001 | 1.95 (1.68-2.26) | <0.001 | 0.73 (0.66-0.79) | <0.001 | 1.70 (1.59-1.82) | <0.001 |
| No. positive cores |  |  |  |  |  |  |  |  |
| <3 | 1.0 (Ref.) |  | 1.0 (Ref.) |  | 1.0 (Ref.) |  | 1.0 (Ref.) |  |
| ≥3 | 0.51 (0.44-0.59) | <0.001 | 0.50 (0.43-0.58) | <0.001 | 0.61 (0.57-0.66) | <0.001 | 0.61 (0.56-0.65) | <0.001 |
| Race |  |  |  |  |  |  |  |  |
| Non-Hispanic White | 1.0 (Ref.) |  | 1.0 (Ref.) |  | 1.0 (Ref.) |  | 1.0 (Ref.) |  |
| Non-Hispanic Black | 1.17 (0.99-1.38) | 0.066 | 1.18 (0.97-1.43) | 0.105 | 1.20 (1.10-1.30) | <0.001 | 1.13 (1.03-1.24) | 0.011 |
| Hispanic | 1.06 (0.83-1.34) | 0.654 | 1.08 (0.84-1.40) | 0.554 | 1.15 (1.03-1.29) | 0.016 | 1.05 (0.93-1.18) | 0.423 |
| Others | 1.54 (1.20-1.98) | 0.001 | 1.38 (1.05-1.82) | 0.020 | 1.12 (0.97-1.30) | 0.134 | 1.03 (0.89-1.21) | 0.668 |
| Insurance status |  |  |  |  |  |  |  |  |
| Insured | 1.0 (Ref.) |  | 1.0 (Ref.) |  | 1.0 (Ref.) |  | 1.0 (Ref.) |  |
| Medicaid | 1.14 (0.85-1.52) | 0.383 | 0.79 (0.57-1.09) | 0.155 | 1.38 (1.22-1.56) | <0.001 | 1.08 (0.95-1.23) | 0.224 |
| Uninsured | 0.86 (0.47-1.58) | 0.634 | 0.78 (0.41-1.48) | 0.452 | 1.56 (1.27-1.90) | <0.001 | 1.40 (1.14-1.72) | 0.001 |
| Residency |  |  |  |  |  |  |  |  |
| Urban | 1.0 (Ref.) |  | 1.0 (Ref.) |  | 1.0 (Ref.) |  | 1.0 (Ref.) |  |
| Rural | 1.03 (0.81-1.31) | 0.826 | 1.01 (0.77-1.33) | 0.946 | 1.04 (0.92-1.18) | 0.53 | 1.04 (0.91-1.18) | 0.597 |
| Marital status |  |  |  |  |  |  |  |  |
| Married | 1.0 (Ref.) |  | 1.0 (Ref.) |  | 1.0 (Ref.) |  | 1.0 (Ref.) |  |
| Single | 1.68 (1.41-2.02) | <0.001 | 1.89 (1.55-2.30) | <0.001 | 1.65 (1.51-1.81) | <0.001 | 1.61 (1.46-1.76) | <0.001 |
| Divorced | 1.71 (1.39-2.11) | <0.001 | 1.82 (1.45-2.30) | <0.001 | 1.63 (1.46-1.81) | <0.001 | 1.62 (1.45-1.80) | <0.001 |
| Widowed | 2.13 (1.60-2.82) | <0.001 | 1.36 (0.99-1.87) | 0.061 | 2.08 (1.82-2.36) | <0.001 | 1.52 (1.33-1.74) | <0.001 |
| Separated | 2.34 (1.43-3.84) | 0.001 | 2.70 (1.60-4.56) | <0.001 | 2.05 (1.64-2.56) | <0.001 | 2.04 (1.62-2.56) | <0.001 |
| SES status |  |  |  |  |  |  |  |  |
| Low tertile | 1.0 (Ref.) |  | 1.0 (Ref.) |  | 1.0 (Ref.) |  | 1.0 (Ref.) |  |
| Middle tertile | 1.00 (0.84-1.18) | 0.972 | 1.12 (0.93-1.35) | 0.236 | 0.91 (0.83-0.99) | 0.022 | 1.02 (0.94-1.12) | 0.595 |
| High tertile | 0.89 (0.76-1.05) | 0.163 | 1.09 (0.89-1.32) | 0.414 | 0.84 (0.78-0.92) | <0.001 | 1.04 (0.95-1.14) | 0.424 |

Abbreviation: AS/WW, active surveillance/watchful waiting; RT, radiation therapy; NCCN, National Comprehensive Cancer Network; OR, odds ratio; PSA, prostate-specific antigen; Ref, reference; SES, socioeconomic status.
